# Stable and null current hysteresis perovskite solar cells based nitrogen doped graphene oxide nanoribbons hole transport layer

**DOI:** 10.1038/srep27773

**Published:** 2016-06-09

**Authors:** Jeongmo Kim, Mohd Asri Mat Teridi, Abd. Rashid bin Mohd Yusoff, Jin Jang

**Affiliations:** 1 Department of Information Display, Kyung Hee University, Dongdaemoo-gu, 130-701 Seoul, Korea; 2 Solar Energy Research Institute, National University of Malaysia, 43600 Bangi, Selangor, Malaysia

## Abstract

Perovskite solar cells are becoming one of the leading technologies to reduce our dependency on traditional power sources. However, the frequently used component poly(3,4-ethylenedioxythiophene) polystyrene sulfonate (PEDOT:PSS) has several shortcomings, such as an easily corroded indium-tin-oxide (ITO) interface at elevated temperatures and induced electrical inhomogeneity. Herein, we propose solution-processed nitrogen-doped graphene oxide nanoribbons (NGONRs) as a hole transport layer (HTL) in perovskite solar cells, replacing the conducting polymer PEDOT:PSS. The conversion efficiency of NGONR-based perovskite solar cells has outperformed a control device constructed using PEDOT:PSS. Moreover, our proposed NGONR-based devices also demonstrate a negligible current hysteresis along with improved stability. This work provides an effective route for substituting PEDOT:PSS as the effective HTL.

Perovskite solar cells have attracted extraordinarily wide attention due to their excellent power conversion efficiency (PCE) values, up to 20.1%, as reported by Sang *et al.*^1^. The advantages of perovskite solar cells include their ability to be fabricated using low-cost and low-temperature solution-based processing, high optical absorption, light weight, and potential to be manufactured on large-area flexible substrates. Although the PCE of perovskite solar cells is comparable to that of thin-film or silicon-based solar cells, it is better than that of other solar cell technologies, such as the dye-sensitized solar cells, organic solar cells, and solar cells with extremely thin absorber layers.

Despite their excellent PCE values, perovskite solar cells have a stability problem and thus tend to degrade faster than other types of solar cells. One probable origin of this problem is the formation of a poor interface between the substrate and the hole or electron collection layer, which subsequently influences the efficiency and stability of the perovskite solar cells^2^. In perovskite solar cells, poly(3,4-ethylenedioxythiophene) polystyrene sulfonate (PEDOT:PSS)^3^,^4^, has been generally used to improve the anode contact for superior hole collection. One advantage of PEDOT:PSS is that it can be deposited from solution, which results in the planarization of the glass substrate roughness and consequently the minimization of its detrimental effects. However, perovskite solar cells based on PEDOT:PSS as the HTL suffer from poor long-term stability because the sensitivity of the PEDOT:PSS to high acidity results in hygroscopic properties and inhomogeneous electrical properties^5^,^6^.

Recently, nanocomposite graphene oxide (GO) has been introduced into perovskite solar cells as a HTL by Wu *et al.* and Wang *et al.*^7^,^8^. Perovskite films grown on GO exhibit enhanced crystallization, high surface area and preferred in-plane orientation of the (110) plane. This approach indicates that graphene–based derivatives have significant potential to influence device performance and thus enhance the PCE of perovskite solar cells. To date, numerous dopants have been used to reduce sheet resistance without sacrificing transmittance. Therefore, it is crucial to search for a suitable dopant to improve conductivity without deteriorating transmittance. The objectives of the present work are i) to propose an alternative material to the PEDOT:PSS layer, which will not have an effect on the ITO or FTO electrodes, and ii) to propose a new dopant for GO nanoribbons and subsequently improve their optical and electrical properties as well as the device performance.

In this work, nitrogen-doped GO nanoribbon (NGONR) films on the FTO substrate are used as the HTL in the perovskite structure: FTO/NGONR/CH_3_NH_3_PbI_3_/ZnO NPs/Al. The effect of the NGONRs on the PCE of the cell has been investigated. The standard FTO/PEDOT:PSS/CH_3_NH_3_PbI_3_/ZnO NPs/Al structure is also fabricated and examined for comparison. The effects of the NGONRs on the morphology, hysteresis and lifetime of the perovskite solar cell are also investigated. The N dopant is chosen to introduce additional n-type carriers to the carbon systems. Throughout this manuscript, Device A is the designation for PEDOT:PSS, Device B for NGONRs, Device C for GO and Device D for GONRs.

## Results and Discussion

**Figure 1:**
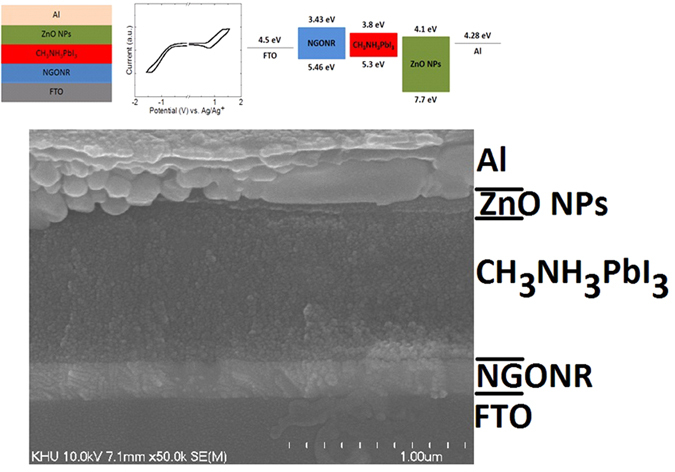
(**a**) Device schematic diagram of our proposed CH_3_NH_3_PbI_3_ cell, (**b**) cyclic voltammograms of NGNOR, and (**c**) energy levels diagram with NGONR hole extraction layer.

Figure 1a illustrates the device schematic diagram of perovskite solar cells utilizing NGONRs and zinc oxide nanoparticles (ZnO NPs) as the hole and electron transport materials (H/ETM), respectively. The complete device configuration is: FTO/HTL (PEDOT:PSS or NGONR)/CH_3_NH_3_PbI_3_/ZnO NPs/Al. From cyclic voltammetry (CV) measurements (Fig. 1b), we determined the highest occupied molecular orbital (HOMO) and the lowest unoccupied molecular orbital (LUMO) levels of NGONR to be 5.46 and 3.43 eV, respectively. Additional data associated with the HOMO and LUMO energy levels of the NGONRs can be found in the Supplementary Information.

The obtained values are perfectly matched with the valence (VB) and conduction bands (CB) of the CH_3_NH_3_PbI_3_ absorber layer (VB: 5.3 and CB: 3.8 eV), respectively. Thus, we anticipate a low energy barrier for hole transport from CH_3_NH_3_PbI_3_ to FTO that also serves to block electron back diffusion to the FTO side (Fig. 1c). ZnO NPs are employed as the electron transport layer because of their well-known efficient electron transport/hole blocking capability from CH_3_NH_3_PbI_3_ to the Al. Figure 1d illustrates a cross-sectional transmission electron microscope image of our fabricated devices. Figure S1 illustrates the NGONRs with different magnifications.

**Figure 2:**
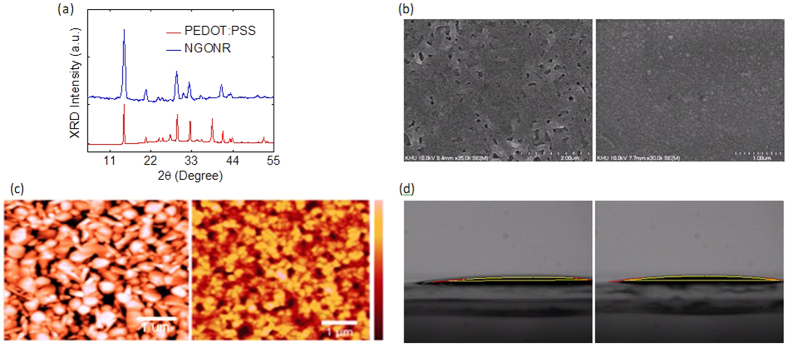
(**a**) XRD patterns of CH_3_NH_3_PbI_3_ films on PEDOT:PSS and NGONR layers. (**b**) SEM images of CH_3_NH_3_PbI_3_ films spin-coated on top of (left) PEDOT:PSS and (right) NGONR. Scale bar is 2 μm. (**c**) AFM images of CH_3_NH_3_PbI_3_ films on (right) PEDOT:PSS and (left) NGONR. Scale bar is 1 μm. (**d**) Contact angles of (left) PEDOT:PSS and (right) NGONR films.

To probe the influence of the PEDOT:PSS and NGONR layers on CH_3_NH_3_PbI_3_, X-ray diffraction (XRD) measurements were carried out. CH_3_NH_3_PbI_3_ films were spin-coated onto glass substrates coated with PEDOT:PSS and NGONR. Both films exhibit diffraction peaks at 14.15°, 28.47° and 43.12°, corresponding to the (110), (220) and (314) planes of the tetragonal perovskite phase (Fig. 2a). The observed peaks are in agreement with the previously reported XRD data^9^,^10^,^11^. Other important figures of merit in perovskite solar cells are the surface coverage and the CH_3_NH_3_PbI_3_ morphology^12^,^13^. Scanning electron microscopy (SEM) and atomic force microscopy (AFM) images were taken to further understand the CH_3_NH_3_PbI_3_ morphology after being deposited on top of the PEDOT:PSS and NGONR layers. As shown in Fig. 2b, a CH_3_NH_3_PbI_3_ layer spin-coated on top of PEDOT:PSS constitutes an incomplete, inhomogeneous and less dense film that is characterized by small voids. This undesirable morphology is further supported by the AFM image that shows the root mean square (rms) roughness of the perovskite layer to be approximately 3 nm (Fig. 2c). In contrast, the SEM image of the CH_3_NH_3_PbI_3_ layer that is deposited on top of the NGONR layer is found to be highly dense and smooth without the presence of voids and to exhibit the rms roughness value of approximately 1 nm (Fig. 2b). Moreover, our findings are further supported by the contact angle data, in which both layers demonstrate low contact angles (Fig. 2d). As the above-mentioned conditions (wettability and surface coverage, and hydrophilicity), are desirable for the perovskite solution, high performance of perovskite solar cells can be obtained if these are met.

**Figure 3:**
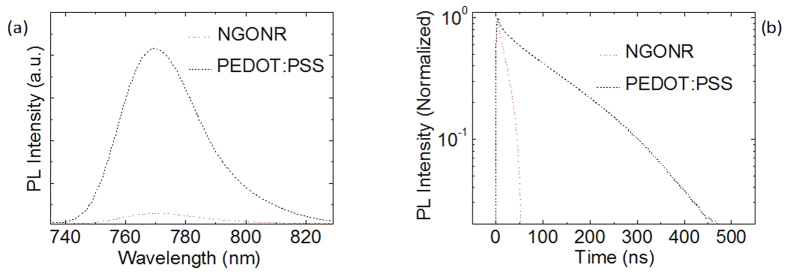
(**a**) Steady-state PL spectra and (**b**) time-resolved PL decay transients of CH_3_NH_3_PbI_3_ films on PEDOT:PSS and NGONR layers. PL decay transients were collected at 770 nm for all films in vacuum after excitation at 405 nm.

**Figure 4:**
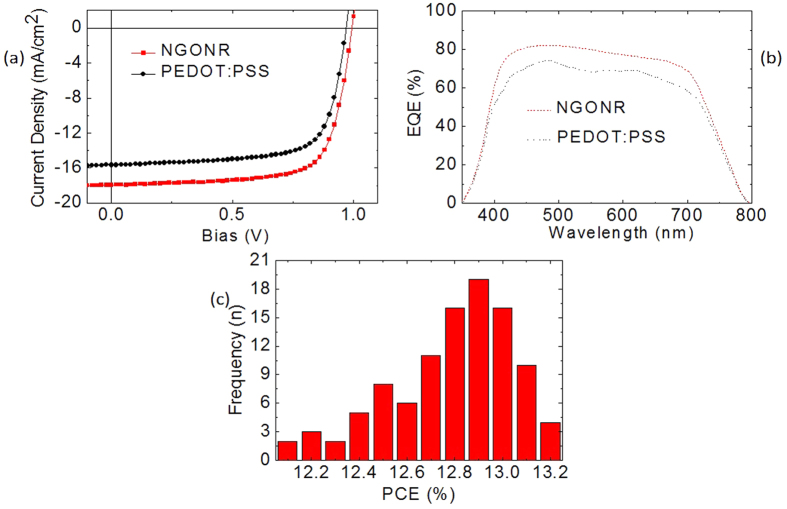
(**a**) J-V curves of the devices with two different HTMs under 1 sun illumination (100 mW/cm^2^). (**b**) External quantum efficiency of the devices. (**c**) The distribution of fabricated devices.

Next, steady-state photoluminescence (PL) and time-resolved PL decay measurements were conducted to gain a deep understanding of the extracted free carrier concentration and dissociation of excitons into free charges. As shown in Fig. 3a, a significant quenching effect occurs on glass/NGONR/CH_3_NH_3_PbI_3_ compared to glass/PEDOT:PSS/CH_3_NH_3_PbI_3_, where the PL quenching for glass/NGONR/CH_3_NH_3_PbI_3_ is approximately 70% less than that of glass/PEDOT:PSS/CH_3_NH_3_PbI_3_. The stronger quenching effect observed in NGONRs is due to a higher hole accepting density of states, thereby affording more efficient hole extraction. In addition, Fig. 3b demonstrates the time-resolved PL decay rate of the PEDOT:PSS and NGONR layers. A PL lifetime is defined as the time it takes for the PL intensity to fall to 1/e from its initial intensity. The average PL decay rate for glass/NGONR/CH_3_NH_3_PbI_3_ is significantly reduced to 22 ns compared to glass/PEDOT:PSS/CH_3_NH_3_PbI_3_ (83 ns). This phenomenon indicated that the holes, which are separated from the generated excitons within the CH_3_NH_3_PbI_3_, are efficiently extracted into the NGONRs. From the PL quenching and the time-resolved PL decay measurements, it is confirmed that the NGONRs can not only extract but also transport holes from the CH_3_NH_3_PbI_3_ to the FTO side. Figure 4a illustrates the J-V characteristics of the average perovskite solar cells employing PEDOT:PSS and NGONR layers. Device A demonstrates a short-circuit current density (J_SC_) of 15.65 mA/cm^2^, an open-circuit voltage (V_OC_) of 0.96 V, a fill factor (FF) of 72.28% and a PCE of 10.86%. A remarkable improvement is observed in the device utilizing NGONRs as the HTL (Device B), where the J_SC_ improves from 15.65 to 17.93 mA/cm^2^, the V_OC_ is 1 V, and the FF is 72.16%, leading to a PCE of 12.94%. The photovoltaic parameters are tabulated in Table 1, including series resistance (R_S_) and J_SC_ calculated from the EQE spectra (Fig. 4b). The integrated J_SC_ values for both devices are in agreement with the J_SC_ values obtained from the J-V characteristics. The morphology of the perovskite film possessing good uniformity and high coverage plays an important role in the device performance. Figure 2b,c illustrate the morphology of perovskite films that were deposited onto different substrates (PEDOT:PSS and NGONRs). The deposition of perovskite films onto the NGONRs allows the perovskite film to grow into large textured domains, yielding an almost complete coverage. Unlike the NGONRs/perovskite interface, the deposition of a perovskite film on top of the PEDOT:PSS leads to some voids, which, in turn, result in lower surface coverage. As a reference, GO- (Device C) and GONR- (Device D) based perovskite solar cells are fabricated using the same device structure. Device fabrication details can be found in the Experimental Section. In these reference measurements, Device D demonstrates a slightly better performance compared to that of Device C. Device D exhibits a J_sc_ of 17.42 mA/cm^2^, V_oc_ of 1 V, FF of 71.25% and PCE of 12.41%, whilst Device C demonstrates a J_sc_ of 15.72 mA/cm^2^, V_oc_ of 1 V, FF of 61.72% and PCE of 9.70% (see Data in Brief). The J_sc_ values, which are determined by integrating the EQE curve with an AM 1.5G reference spectrum, are consistent with the obtained J_sc_ values from the J-V characteristics (see Data in Brief). The N dopant does not appear to influence the solar cells’ performance (Device B and Device D). We are, therefore, currently pursuing further investigation to gain a better understanding of the optical and electrical properties of doped and undoped graphene and solar cells parameters.

To understand the improved performance by utilizing NGONRs, we conducted optical transmittance measurements on PEDOT:PSS and NGONR films. The transmittance of PEDOT:PSS films is lower compared to that of the NGONR films (data not shown). Thus, we conclude that higher transparency improves light harvesting in Device B, which in turn enhances the J_sc_ and subsequently the solar efficiency. In addition, the energy band diagram also illustrates that the enhanced device performance can be attributed to the improved hole extraction and electron blocking capabilities as well as the uniform surface morphology, almost complete surface coverage and the presence of fewer pinholes in the CH_3_NH_3_PbI_3_ film deposited on the NGONR layer.

In a separate experiment, bottom contact organic field effect transistors were fabricated to demonstrate the hole mobility of the NGONRs, which we extracted from the linear region to be 9 × 10^−3^ cm/V s. Thus, the high performance of Device B may stem from the well-matched energy alignment and high mobility of the NGONRs.

To elucidate the reproducibility of the fabrication process, 102 devices were fabricated using the same process and parameters. The photovoltaic parameters of these devices are shown in Fig. 4c, together with the corresponding standard deviations, which are summarized in Table 1. The devices of each HTL demonstrate consistent photovoltaic performance, as proven by the rather small standard deviations of the performance parameters.

**Figure 5:**
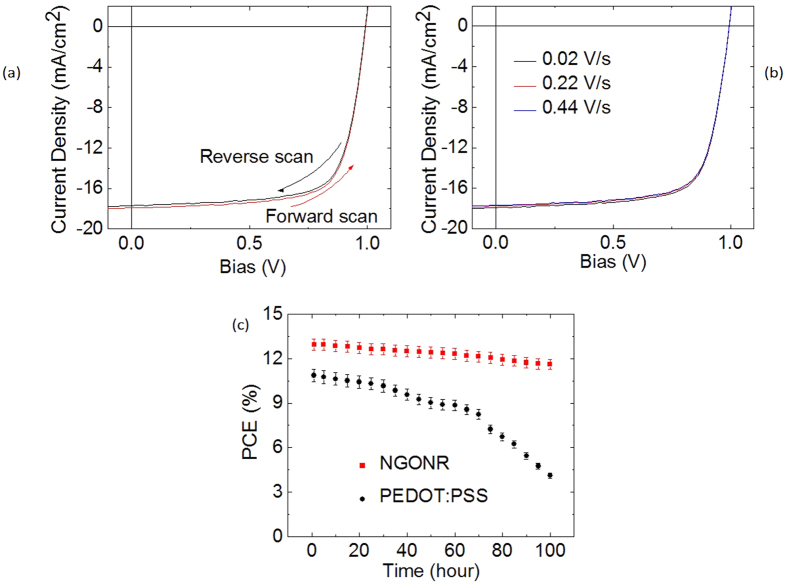
J-V scans of the CH_3_NH_3_PbI_3_ solar cell with NGONR HTM (**a**) different scanning directions, (**b**) different voltage sweep rates under 1 sun illumination (100 mW/cm^2^) demonstrating null current hysteresis, and (**c**) stability of encapsulated perovskite solar cell with PEDOT:PSS and NGONR HTMs over 100 hours under ambient conditions.

Currently, many reported works on perovskite solar cells have demonstrated negligible current hysteresis at various voltage scanning rates or scan directions (Fig. 5). In general, the observed current hysteresis can be attributed to i) slow dynamic processes due to the charge trapping and de-trapping because of the low quality of perovskite films, ii) an unbalanced electron and hole transport rate, and iii) defects in the interface^14^,^15^. Accordingly, the operation of a perovskite solar cell must not be judged solely on the J-V characteristics, but rather on the constant power output at maximum power point conditions. Figure 5 shows the J-V characteristics of the CH_3_NH_3_PbI_3_ solar cell prepared with NGONRs as the HTL scanned in two different directions (forward and reverse scans), and various scan rates (0.02, 0.22, and 0.44 V/s) with a delay time of 40 ms. They demonstrate negligible current hysteresis. Our findings imply that current hysteresis is perhaps due to defects in the CH_3_NH_3_PbI_3_ film and poor interfaces in the FTO/NGONR/CH_3_NH_3_PbI_3_ structure, which can be partly solved by using a high-quality CH_3_NH_3_PbI_3_ film prepared through a two-step deposition. In addition, the negligible current hysteresis in our study indicates that the fabrication approach demonstrated here is sufficient to avoid such behavior. It is accepted that the two-step deposition is the most promising method to acquire highly crystalline, homogeneous, smooth, dense and continuous perovskite films. In addition, the quality of the CH_3_NH_3_PbI_3_ film depends on the quality of the PbI_2_ film. Accordingly, this is what motivated us to implement a straightforward device architecture; attaining a highly dense and smooth PbI_2_ layer on the surface of the PEDOT:PSS layer is easier compared to depositing a CH_3_NH_3_PbI_3_ layer because PbI_2_ is less ionic than MAPbI_3_. A smooth, highly dense and high quality PbI_2_ film can be achieved by adding a small amount of water to the precursor solution.

Finally, we tested the lifetime of CH_3_NH_3_PbI_3_ solar cells using different HTLs under ambient atmosphere. The average temperature and humidity values are 20 °C and 47%, respectively. We found that degradation happens only in CH_3_NH_3_PbI_3_ solar cells utilizing PEDOT:PSS as the HTL after 5 h of exposure to air (Fig. 5c). Although the film on NGONR starts to degrade after 50 h, its degradation rate is slower than that of the film on PEDOT:PSS. The efficiency of the CH_3_NH_3_PbI_3_ solar cell with PEDOT:PSS as the HTL is reduced to a PCE of 4.11%, whereas the CH_3_NH_3_PbI_3_ solar cell using NGONR is still above 11% from its initial value. This suggests that the acidic nature of PEDOT:PSS accelerates the degradation of the CH_3_NH_3_PbI_3_ solar cell.

## Conclusions

In summary, we have successfully utilized NGONRs as the HTL in perovskite solar cells. Excellent wetting of perovskite precursor solution on the NGONR layer leads to a uniform active layer film with complete surface coverage and superior hole selectivity for facilitating hole transport from the perovskite to the FTO anode. As a result, the device with NGONRs exhibits a device efficiency over 12%, higher than that of the device fabricated with the widely used PEDOT:PSS. The device fabricated with NGONRs also demonstrates negligible current hysteresis regardless of scanning direction and sweeping rates. Furthermore, NGONRs improve the device stability in air compared to PEDOT:PSS.

## Experimental

### Materials

Multiwalled carbon nanotubes, aniline, ammonium persulfate, and commercial Pt/C catalysts (20 wt%) used in this study were purchased from Sigma Aldrich.

### Preparation of NGONR

NGONR was prepared according to the previously modified published^1^ work by pyrolyzing GONR/PANI composites at 900 °C for 1 h in an argon atmosphere.

### Preparation of GO and GONRs

The GO and GONRs were prepared according to the previously published works^2^,^3^,^4^.

### Solar cell fabrication

Solar cells were fabricated on pre cleaned FTO-coated glass substrates with a sheet resistance of 20 Ω/sq. First, a thin PEDOT:PSS layer was spin coated onto the substrate at 4000 rpm for 30 s and baked at 120 °C for 10 min. In the case of NGONRs- and GONRs based devices, the NGONRs were deposited at 2500 rpm for 30 s followed by annealing at 120 °C for 1 h. For GO-based devices, the GO layer was deposited at 1500 rpm for 30 s followed by annealing at 120 °C for 1 h. A PbI_2_ solution (dissolved in N,N-dimethylformamide at a concentration of 460 mg/ml) was then spin coated on top of the PEDOT:PSS layer at 3000 rpm for 15 s. After drying for several minutes in air, the substrate was dipped into a solution of CH_3_NH_3_I in 2-propanol (10 mg/ml) for 40 s, then dried under a flow of clean air. Later, the ZnO NPs were spin-coated at 4000 rpm on the perovskite layer and annealed at 80 °C for 60 min to form a 20 nm condensed layer. The ZnO NPs was prepared using the hydrothermal method. To complete the fabrication procedure, a 150-nm-thick aluminium layer was deposited by thermal evaporation at a base pressure of 1 × 10^−7^ Torr. Prior to any electrical measurements, all devices were encapsulated with glass slides. All our fabricated devices had 0.1 cm^2^ active area.

### Solar Cell Measurements

The performance of the perovskite solar cells was obtained from J-V characteristics measured using a Keithley 2400 LV source meter. Solar cell performance was measured using a solar simulator, with an Air Mass 1.5 Global (AM 1.5 G) and had an irradiation intensity of 100 mW/cm^2^. All measurements were carried out at room temperature, under a relative humidity of 47%. The EQE measurements were performed using the EQE system (Model 74000) obtained from Newport Oriel Instruments USA and HAMAMATSU calibrated silicon cell photodiodes as a reference diode. The wavelength was controlled with a monochromator of 200–1600 nm.

### Transient photocurrent decay measurements

Time resolved photoluminescence measurements were conducted using a Hamamatsu streak camera containing a photocathode sensitized from the visible spectrum to 1300 nm and operated in the synchroscan mode. The samples were excited at 425 nm by the frequency-doubled output of a mode locked Ti:sapphire laser.

### Scanning electron microscopy

The scanning was conducted using a SEM, Hitachi S-4700.

### Atomic force microscopy

The surface morphology of the thin films was obtained by tapping mode using an atomic force microscope (AFM, Digital Instrument Multimode equipped with a nanoscope IIIa controller).

### Thickness measurements

The film thickness was measured using a Dektak AlphaStep Profiler.

### XRD measurements

The X-ray diffraction (XRD) analysis was performed using a XRD Diffractometer X’Pert PRO with Cu Kα targets (λ = 0.154 nm) at a scanning rate of 2°/min and an operating voltage of 40 kV with a current of 100 mA.

### Contact angle measurements

Contact angle measurement was performed using an Attension Theta optical tensiometer with automated liquid pumping system was used for the contact angle measurements.

### Degradation study

Stability measurements were carried out with a periodically exposure to AM1.5G conditions, 100 mW/m^2^ and the J-V characteristics were recorded every 5 hours up to 100 hours according to the ISOS-L-1 procedure^5^.

### HOMO and LUMO determination

According to the onset potential of the reduction and oxidation process, the LUMO and HOMO energy level of NGONRs can be calculated using eq. 1 and 2


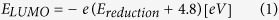



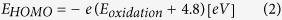


The calculated HOMO is found to be 5.46 eV and LUMO is approximately 3.43 eV leading to the electrochemical bandgap of NGONRs about 2.03 eV.

## Additional Information

**How to cite this article**: Kim, J. *et al.* Stable and null current hysteresis perovskite solar cells based nitrogen doped graphene oxide nanoribbons hole transport layer. *Sci. Rep.* **6**, 27773; doi: 10.1038/srep27773 (2016).

## Supplementary Material

Supplementary Information

## Figures and Tables

**Table 1:** The photovoltaic parameters of FTO/HTM/CH_3_NH_3_PbI_3_/ZnO NPs/Al solar cells under 100 mW/cm^2^ AM1.5 illumination.

HTM	J_SC_ (mA/cm^2^)	aJ_SC_ (mA/cm^2^)	V_OC_ (V)	FF (%)	PCE (%)	R_S_(Ω cm^2^)
PEDOT:PSS	15.65 ± 0.11	15.32	0.96 ± 0.01	72.28 ± 0.13	10.86 ± 0.07	5.83
NGONR	17.93 ± 0.07	17.34	1.00 ± 0.01	72.16 ± 0.09	12.94 ± 0.04	5.67

^a^Calculated from EQE spectra.
