# Supplementary material for: Stable and null current hysteresis perovskite solar cells based nitrogen doped graphene oxide nanoribbons hole transport layer
Source: Sci Rep. 2016 Jun 9;6:27773. doi: 10.1038/srep27773 (PMC4899776; doi:10.1038/srep27773)
Supplement: Supplementary Information [file srep27773-s1.doc]

Supporting Information

Stable and null current hysteresis perovskite solar cells based nitrogen doped graphene oxide nanoribbons hole transport layer

Jeongmo Kim,^a^ Mohd Asri Mat Teridi,^b^ Abd. Rashid bin Mohd Yusoff^a^ and Jin Jang^a*^

^a^Department of Information Display, Kyung Hee University, Dongdaemoo-gu, 130-701 Seoul, Korea. Email: jjang@khu.ac.kr

^b^Solar Energy Research Institute, National University of Malaysia, 43600 Bangi, Selangor, Malaysia


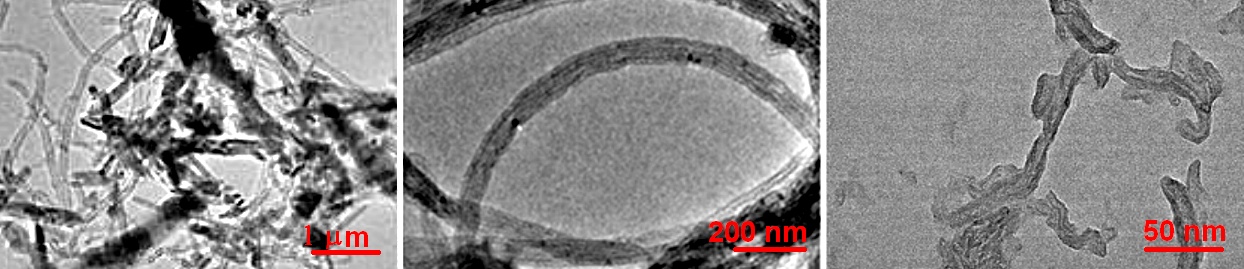


Figure S1. NGONRs with different magnifications.

**Figure S2.** J-V characteristics of the devices with two different HTMs under 1 sun illumination (100 mW/cm^2^).

Figure S3. External quantum efficiency of the devices with two different HTMs.

References

1. Silva, R. C. et al. Formation of Nitrogen-Doped Graphene Nanoribbons via Chemical Unzipping. *ACS Nano* *7*, 2192-2204 (2013).
2. Hummers, W. S.; Offeman, R. E. Preparation of Graphitic Oxide. *J. Am. Chem. Soc.* ***80***, 1339-1339 (1958).
3. Liu, J.; Xue, Y.; Dai, L. Sulfated Graphene Oxide as A Hole-Extraction Layer in High Performance Polymer Solar Cells. *J. Phys. Chem. Lett.* ***3***, 1928-1933 (2012).

4 Li, S. S. et al. Solution-Processable Graphene Oxide as an Efficient Hole Transport Layer in Polymer Solar Cells. *ACS Nano* *4*, 3169-3174 (2010).

5 Resse, M.O. et al. Consensus Stability Testing Protocols for Organic Photovoltaic Materials and Devices. *Sol. Energy Mater. Sol. Cells* 95, 1253-1267 (2011).
